# Analysis of the Transmission of SARS-CoV-2 Delta VOC in Yantai, China, August 2021

**DOI:** 10.3389/fmed.2022.842719

**Published:** 2022-05-30

**Authors:** Yulou Sun, Yuwei Zhang, Zimo Liu, Xia Li, Juan Liu, Xinghan Tian, Qiao Gao, Peihua Niu, Hongli Zhai, Zhenlu Sun, Yunlong Tian, Ji Wang

**Affiliations:** ^1^Yantai Center for Disease Control and Prevention, Yantai, China; ^2^Shandong Center for Disease Control and Prevention, Jinan, China; ^3^Electrocardiogram Room, Yantai Yuhuangding Hospital, Yantai, China; ^4^Department of Critical Care Medicine, Yantai Yuhuangding Hospital Affiliated to Qingdao University, Yantai, China; ^5^National Institute for Viral Disease Control and Prevention, Chinese Center for Disease Control and Prevention, Beijing, China; ^6^Research Institute of Luye Public Health, Ludong University, Yantai, China; ^7^Chinese Field Epidemiology Training Program, Chinese Center for Disease Control and Prevention, Beijing, China

**Keywords:** analysis, transmission, SARS-CoV-2 Delta, Yantai, China

## Abstract

**Objective:**

Starting 31 July 2021, a SARS-CoV-2 outbreak occurred in Yantai, Shandong Province. The investigation showed that this outbreak was closely related to the epidemic at Nanjing Lukou Airport. In view of the fact that there were many people involved in this outbreak and these people had a complex activity area, the transmission route cannot be analyzed by simple epidemiological investigation. Here we combined the SARS-COV-2 whole-genome sequencing with epidemiology to determine the epidemic transmission route of Yantai.

**Methods:**

Thirteen samples of SARS-CoV-2 outbreak cases from 31 July to 4 August 2021 were collected and identified by fluorescence quantitative PCR, then whole-genome deep sequencing based on NGS was performed, and the data were analyzed and processed by biological software.

**Results:**

All sequences were over 29,000 bases in length and all belonged to B.1.617.2, which was the Delta strain. All sequences shared two amino acid deletions and 9 amino acid mutations in Spike protein compared with reference sequence NC_045512.2 (Wuhan virus strain). Compared with the sequence of Lukou Airport Delta strain, the homology was 99.99%. In order to confirm the transmission relationship between patients, we performed a phylogenetic tree analysis. The results showed that patient 1, patient 2, and patient 9 belong to an independent branch, and other patients have a close relationship. Combined with the epidemiological investigation, we speculated that the epidemic of Yantai was transmitted by two routes at the same time. Based on this information, our prevention and control work was carried out in two ways and effectively prevented the further spread of this epidemic.

## Introduction

SARS-COV-2 is a novel coronavirus first reported in Wuhan, China in December 2019 which caused an epidemic of acute respiratory syndrome (1, 2). Since then, the coronavirus disease 2019 (COVID-19) has spread quickly all over the world causing great casualties and property losses (3, 4). By mid-March 2022, nearly 460 million cases of COVID-19 were diagnosed with over 6 million deaths around the world (https://coronavirus.jhu.edu/map.html). All viruses, including SARS-CoV-2, change over time. Most changes have little to no effect on virus properties, but some changes especially the mutation accumulation may affect the propagation, pathogenicity, performance of vaccines, diagnostic tools, and so on (5). In order to prioritize global monitoring and research, and ultimately inform the ongoing response to the COVID-19 pandemic, the world health organization (WHO) classified important variants into two categories: variants of concern (VOC) and variants of interest (VOI) (https://www.who.int/en/activities/tracking-SARS-CoV-2-variants). VOC means that the Virus strains have a wide range of influence, and data supports it enhancing the transmissibility and detrimental change or reduces the vaccine effectiveness and therapeutic effect. VOI means that the Virus strains are predicted or known to change characteristics, and have been found in many countries with an increasing number of cases over time. Given the continuous evolution of the virus and the constant developments in our understanding of the impacts of variants, these definitions may be periodically adjusted. Currently, there are five designated VOCs (Alpha from the UK, Beta from South Africa, Gamma from Brazil, Delta from India, and Omicron from Multiple countries) and two VOIs (Lambda from Peru and Mu from Colombia). Each strain contains its unique characteristic mutation spectrum and also has the same mutation sites among strains. Alpha, Beta, and Gamma have the same mutation N501Y within the receptor-binding domain (RBD) of the spike protein, which can increase the affinity to human angiotensin-converting enzyme 2 (hACE2) (6, 7). This may play an essential role in the higher transmission of these strains. Beta and Gamma have another shared mutation, E484K, in their spike protein, this mutation can not only enhance the receptor binding affinity but also can escape the neutralization by vaccine-induced humoral immunity or some therapeutic monoclonal antibodies (8–10). Focusing on the mutations of the Delta strain, it hosts L452R T478K P681R mutations in RBD, these can greatly improve the transmission ability and immune system evasion (11, 12). Since April 2021, Delta has expanded rapidly in the world until the emergence of Omicron in December 2021. Omicron contains more than 15 mutations in RBD, these mutations greatly changed the structure of Spike protein, enhanced its binding ability to ACE2, and invalidated many antibody binding sites (13, 14). In addition, Omicron also got rid of the dependence on cellular protease TMPRSS2 and made it reproduce rapidly and massively in airway cells above the lungs that do not express TMPRSS2, which not only increased the viral load but also accelerated the transmission speed of the virus (15, 16). At present, Omicron has almost completely replaced Delta all over the world.

China was also troubled by the SARS-COV-2 Delta strain. Since June 2021, it has been found in new outbreaks in Yunnan, Guangdong, and Jiangsu. The outbreak started at Lukou Airport of Nanjing with related epidemics in many provinces and cities. Because of omissions in cleaning and disinfection of an inbound Russian aircraft CA910 which arrived at Lukou Airport in Nanjing from Moscow on July 10, the cleaning staff were infected with SARS-COV-2 and then caused the spread of the infection. The investigation showed that the SARS-COV-2 outbreak in Yantai was also closely related to this source. The first Lukou-related case in Yantai was diagnosed on 31 July 2021 and a total of 13 patients were finally diagnosed in 5 days. It was worth noting that the epidemiological investigation showed that the transmission relationship among the 13 people was complex. So in order to determine the virus strains type and the transmission relationship between cases, we sequenced the whole genomic nucleic acids of these 13 cases based on second-generation high-throughput sequencing technology (NGS), analyzed the gene characteristics and variation of the virus from the molecular level, and traced the source of the virus.

## Methods and Materials

### Sample Collection

Since 31 July 2021, a SARS-CoV-2 outbreak has occurred in Yantai, Shandong Province. As of 4 August, a total of 13 novel coronavirus-positive cases have been detected. Their nasopharyngeal swabs were collected to our laboratory for testing before sending them to an infectious disease hospital for treatment.

### SARS-CoV-2 Nucleic Acid Diagnosis

Viral RNA was extracted from 140 μL clinical specimens using a QIAamp viral RNA mini kit (Qiagen, Hilden, Germany) following the manufacturer's protocol. The purified RNA was eluted in a 50 μL elution buffer. Fluorescent qPCR was performed using an *In Vitro* Diagnostic (IVD) reagent (Bioperfectus Technologies, Jiangsu, China) prior to sequencing of the PCR product. Open Reading Frame gene region (ORF1a/b), Nucleocapsid region (N) of SARS-CoV-2, and a positive reference gene were used to evaluate the presence and the quantity of SARS-COV-2. We followed kit instructions with thermocycler protocol: 1 cycle 50°C 10 min; 1 cycle 97°C 1 min; 45 cycles 97°C 5 s; 58°C 30 s with fluorescence reading. The circulation threshold (Ct) detection limit was 40 (350 copies/ml). A Ct value <37 is considered positive. All samples' Ct values were <30, meaning that subsequent sequencing steps could be carried out. All tests were conducted under strict biosafety conditions and standard operating procedures.

### Sequencing Strategies

In order to obtain the sequence of SARS-COV-2 specifically, an amplicon-based enrichment method was used for sequencing library preparation. Reverse transcription and amplification steps were performed using ULSEN® 2019-nCoV Whole Genome Kit (Micro-Future, Beijing, China). A measure of 16 μL of viral RNA was reverse-transcribed into the first strand of cDNA and the viral genome was amplified by primer pools A and B. The PCR product was purified with AMPure XP beads (Beckman Coulter, Brea, CA) and diluted to 0.2 ng/μL. Paired-end libraries were generated with Nextera XT DNA Library Preparation Kit (Illumina, San Diego, CA) following the reference guide. Samples were multiplexed, using the Nextera XT index kit (Illumina, San Diego, CA). For the quantification and validation of the library, the Qubit 4.0 Fluorometer system (Life Technologies, Carlsbad, CA) and 2100 Bioanalyzer (Agilent Technologies, Santa Clara, CA) were used. Library sequencing was performed on Miseq using MiSeq Reagent Kit v2 (300-cycles; Illumina, San Diego, CA).

### Data Analysis

For raw data, we first calculated the quality of sequencing reads by FastQC software (Babraham Institute, Cambridge, UK), and clean data was generated after removing sequencing adapters, reads containing poly-N and low quality reads by trimmomatic software (17). All downstream analysis was based on high-quality clean data. The reference genome (NC_045512.2) was downloaded directly from NCBI (National Center for Biotechnology Information). Paired-end clean reads were aligned to the reference genome using BWA-MEM v0.7.17 (18). Mapped reads were sorted by name using sambamba v0.6.8 (19). PCR duplications were processed by GATK (Genome Analysis Toolkit) (20) v4.2.0.0. The full length of virus sequences were obtained by ivar v1.3.1 (21), sequencing depth <3, and uncovered areas were replaced with “N.” For clade assignment and mutation calling, we imported all sequences into Nextclade (https://clades.nextstrain.org/tree) and the web-application Phylogenetic Assignment of Named Global Outbreak Lineages (pangolin: https://pangolin.cog-uk.io/). The full-length SARS-CoV-2 genome sequences were aligned using ClustalW integrated in the MEGA X. The neighbor-joining (NJ) phylogenetic tree was constructed by the program MEGA X using the Kimura two-parameter model and 1,000 bootstrap samplings.

## Results

### Epidemiological History Survey

Patient 1, a native of Laishan District, Yantai, was transferred by plane to Nanjing Lukou Airport on 15 July 2021. He was confirmed as the first positive case of the novel coronavirus outbreak in Yantai on 31 July 2020. Patient 2, a migrant worker in YEDA, was a close contact of patient 1, who was diagnosed with a common case of COVID-19 on 1 August 2021. Patient3, a worker of a beauty salon in YEDA, transshipped at Nanjing Lukou Airport by plane on July 19, 2021. She returned to Yantai on July 22 and was confirmed to be infected with novel coronavirus on August 2. Patient 4, Patient 5, Patient 6, Patient 7, Patient 8, Patient 10, Patient 11, and Patient 13 were all employees of a beauty salon in YEDA and were close contacts of Patient 3. Patient 9 was the wife and close contact of Patient 1. Patient 12 was a close contact of Patient 4 (Table 1).

**Table 1:** Details of patients involved in this study.

**Patient no**.	**Gender**	**Age**	**Date of diagnosis**	**Location**
Patient 1	Male	60	2021.07.31	Laishan district, Yantai
Patient 2	Male	62	2021.08.01	YEDA
Patient 3	Female	39	2021.08.02	YEDA
Patient 4	Male	28	2021.08.02	YEDA
Patient 5	Female	38	2021.08.03	YEDA
Patient 6	Male	29	2021.08.03	YEDA
Patient 7	Female	28	2021.08.03	YEDA
Patient 8	Female	26	2021.08.03	YEDA
Patient 9	Female	57	2021.08.03	Laishan district, Yantai
Patient 10	Female	26	2021.08.04	YEDA
Patient 11	Male	25	2021.08.04	YEDA
Patient 12	Female	33	2021.08.04	YEDA
Patient 13	Male	33	2021.08.04	YEDA

### Nucleic Acid Test Results

Nasopharyngeal swabs were collected from all 13 patients, viral RNA was extracted from a 140 μL sample using a QIAamp viral RNA mini kit (Qiagen, Hilden, Germany) following the manufacturer's protocol. The purified RNA was eluted in a 50 μL elution buffer. Before sequencing, an *In Vitro* Diagnostic (IVD) reagent (Bioperfectus Technologies, Jiangsu, China) applying fluorescent PCR technology was used. Internal quality control was evaluated using a group of positive (confirmed case RNA) and negative (DEPC H_2_O) controls. Results showed that two specific targets (ORF1ab, N gene) of SARS-CoV-2 from 13 cases, standard kit, and positive internal quality control were positive, and an ideal logarithmic curve was obtained.

### Next-Generation Sequencing Results

NGS was used to complete the whole genome sequencing of 13 cases, and a total of 13 novel coronavirus genome sequences were obtained. The fastA sequences of the whole genome were assembled successfully, all of which were over 29,000 bases in length. Through the web-application pangolin, we got the information that the novel coronavirus genome sequences of the above cases all belong to branch B.1.617.2 (Delta) strain.

### Homology Analysis and Gene Traceability Analysis

**Figure 1:**
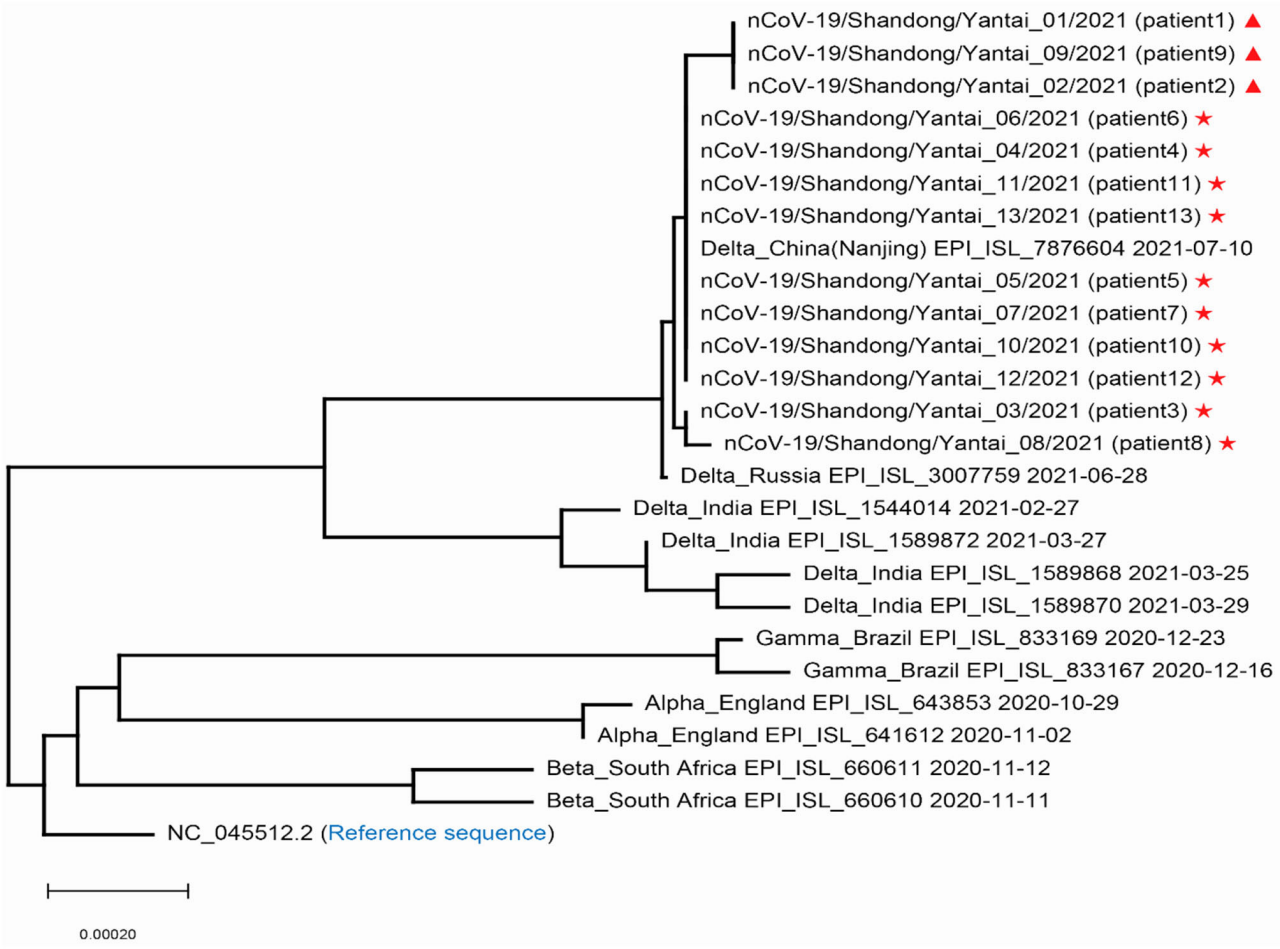
Phylogenetic tree of the SARS-COV-2 sequences in Yantai with other reported sequences. Stars and triangles represent the two transmission routes of COVID-19, respectively in Yantai.

**Figure 2:**
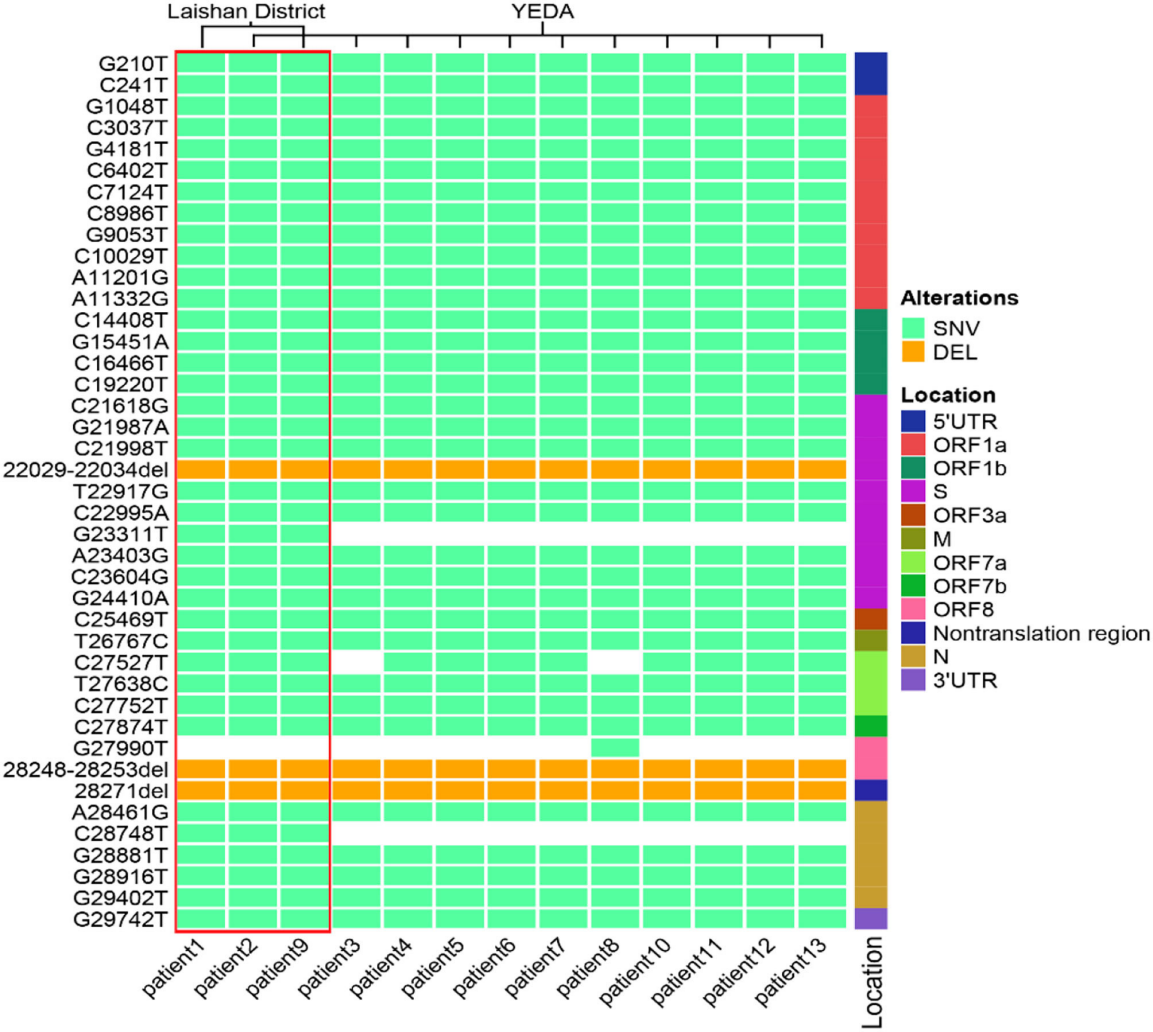
The mutation spectrum of 13 SARS-COV-2 sequences in Yantai. These mutation sites are based on the alignment between the whole genome sequence of the virus and the reference genome. All sites were sorted according to their positions in the genome. The location represents the position of the mutation site in the genome. The pale green box represents SNV, the orange box represents DEL and the white box represents no mutation at this site. The red box contains three patients within the same transmission route.

In order to confirm the close relationship of the epidemic between Yantai and Nanjing Lukou Airport, we aligned our sequences with one SARS-COV-2 sequence from the confirmed case (EPI ISL 7876604) in CA910, and the homology was 99.99%. Due to the fact that the CA910 took off from Moscow, we also aligned our sequences with all SARS-COV-2 sequences from Russia from 20 June to 20 July from the GISAID database. One sequence (EPI_ISL_3007759) collected from Moscow on June 28 2021 was highly homologous with our sequence. This evidence could support that the Yantai epidemic belongs to the transmission chain of the Lukou epidemic. To infer the transmission relationship between all patients, we built a Neighbor-Joining phylogenetic tree based on the whole SARS-COV-2 genome of 13 sequences in Yantai and 12 genomes available on GISAID including the sequence from Russia, and the reference sequence download from NCBI (https://www.ncbi.nlm.nih.gov/sars-cov-2/; Figure 1). Details of all sequences were shown in Table 2. The result showed that patient 1, patient 2, and patient 9 belong to an independent branch, and other patients have a close relationship. Combined with the epidemiological investigation, we speculated that the epidemic in Yantai was transmitted by two routes at the same time. To confirm this speculation, we analyzed their mutation information (Figure 2). A total of 41 mutations were found in the 13 sequences compared to the reference sequence (NC_045512.2). The mutation spectrum of patient 1, patient 2, and patient 9 were the same and included two specific mutations (G23311T in Spike protein, C28748T in N protein), it could be concluded that they belong to the same route and that patient 1 was the source of transmission. There were some differences in the mutations of others, patient 3 had the least number of mutations and had been to Lukou Airport. So patient 3 was the source of transmission of another route, and mutations occurred during passage. Compared with patient 3, patient 8 carried a unique mutation G27990T in ORF8 protein and no other patients had this mutation, which showed that the virus carried by patient 8 had not spread again. Compared with patient 3 and patient 8, patient 4, patient 5, patient 6, patient 7, patient 10, patient 11, patient 12, and patient 13 had the same mutation C27527T in ORF7a protein, we can make sure that the virus had spread between these patients, but we cannot determine the order of transmission.

**Table 2:** Details of all sequences involved in this study.

**Virus name**	**Collection date**	**Location**	**Accession ID**	**Database**	**Note**
hCoV-19/England/QEUH-B12D90/2020	2020.11.02	England	EPI_ISL_641612	GISAID	–
hCoV-19/England/CAMC-B08C45/2020	2020.10.29	England	EPI_ISL_643853	GISAID	
hCoV-19/South Africa/KRISP-EC-K004574/2020	2020.11.11	South Africa	EPI_ISL_660610	GISAID	
hCoV-19/South Africa/KRISP-EC-K004576/2020	2020.11.12	South Africa	EPI_ISL_660611	GISAID	
hCoV-19/India/MH-NCCS-P1162000182735/2021	2021.02.27	India	EPI_ISL_1544014	GISAID	
hCoV-19/India/WB-1931300251103/2021	2021.03.25	India	EPI_ISL_1589868	GISAID	
hCoV-19/India/WB-1931501009078/2021	2021.03.29	India	EPI_ISL_1589870	GISAID	
hCoV-19/India/WB-1931501003695/2021	2021.03.27	India	EPI_ISL_1589872	GISAID	
hCoV-19/Brazil/AM-987/2020	2020.12.16	Brazil	EPI_ISL_833167	GISAID	
hCoV-19/Brazil/AM-989/2020	2020.12.23	Brazil	EPI_ISL_833169	GISAID	
hCoV-19/Russia/MOW-RII-MH27370S/2021	2021.06.28	Russia	EPI_ISL_3007759	GISAID	
hCoV-19/Jiangsu/NJ/2021	2021.07.10	China (Nanjing)	EPI_ISL_7876604	GISAID	
hCoV-19/Shandong/Yantai_01/2021	2021.07.31	Yantai	EPI_ISL_8525417	GISAID	Patient 1
hCoV-19/Shandong/Yantai_02/2021	2021.08.01	Yantai	EPI_ISL_8525418	GISAID	Patient 2
hCoV-19/Shandong/Yantai_03/2021	2021.08.02	Yantai	EPI_ISL_8525419	GISAID	Patient 3
hCoV-19/Shandong/Yantai_04/2021	2021.08.02	Yantai	EPI_ISL_8525420	GISAID	Patient 4
hCoV-19/Shandong/Yantai_05/2021	2021.08.03	Yantai	EPI_ISL_8525421	GISAID	Patient 5
hCoV-19/Shandong/Yantai_06/2021	2021.08.03	Yantai	EPI_ISL_8525422	GISAID	Patient 6
hCoV-19/Shandong/Yantai_07/2021	2021.08.03	Yantai	EPI_ISL_8525423	GISAID	Patient 7
hCoV-19/Shandong/Yantai_08/2021	2021.08.03	Yantai	EPI_ISL_8525424	GISAID	Patient 8
hCoV-19/Shandong/Yantai_09/2021	2021.08.03	Yantai	EPI_ISL_8525425	GISAID	Patient 9
hCoV-19/Shandong/Yantai_10/2021	2021.08.04	Yantai	EPI_ISL_8525426	GISAID	Patient 10
hCoV-19/Shandong/Yantai_11/2021	2021.08.04	Yantai	EPI_ISL_8525427	GISAID	Patient 11
hCoV-19/Shandong/Yantai_12/2021	2021.08.04	Yantai	EPI_ISL_8525428	GISAID	Patient 12
hCoV-19/Shandong/Yantai_13/2021	2021.08.04	Yantai	EPI_ISL_8525429	GISAID	Patient 13
SARS-CoV-2	2019.12	China	NC_045512.2	NCBI	Reference

## Discussion

Delta VOC was first identified in October 2020 and has become a major variant globally since April 2021. According to WHO research, the transmission rate of the Delta virus has increased by nearly 100% compared to other strains not listed as “of concern,” and a recent study of the transmission dynamics of the Delta variant virus that caused the COVID-19 outbreak in Guangdong, China, also suggests that it is twice as infectious as previous pandemic strains (22). The Delta variant also spread faster than other strains. In the past, the incubation period of the Novel Coronavirus has been 5–6 days, and that of the Novel Coronavirus Delta variant is 4 days. The passage interval used to be 4 or 5 days, but now it is about 3 days (23, 24).

Thirteen cases of this outbreak, caused by a Delta variant in Yantai have been locally transmitted. During the study period, the local government implemented an epidemiological follow-up, and we sequenced all confirmed patients. This provides an opportunity for our study to understand its transmission characteristics.

In this outbreak, we found that patient 1 has been to YEDA and infected one close contact, there may be track crossing with other cases in YEDA. So only investigating the track of the action could not determine the transmission relationship of this epidemic. At this time, whole-genome sequence information may provide evidence for genotyping and phylogenetic analysis which help us to resolve this difference (25, 26), of course, this must also be based on a certain basis: their sequences must have enough differences. Fortunately, the virus transmitted this time meets this prerequisite. Through sequence analysis, we determined that this epidemic situation had two transmission routes (Figure 1) and obtained the mutation spectrum (Figure 2) of each virus. Based on this information, the prevention and control work was carried out in two ways immediately and simultaneously. By the end of this epidemic, there were only 13 cases, which was a great achievement for a city with 3 million people. Like other similar studies, it fully illustrates the importance of rapid virus genome analysis in epidemic prevention and control (27–30).

As SARS-CoV-2 continues to spread around the world, the dynamics of virus evolution and mutation are still changing, and new viruses are constantly acquiring new mutations in their genomes. Although some mutations provide the virus with the advantage of resisting human immune response, these mutations may lead to changes in pathogenicity and virulence (31). Therefore, future prevention and control work should strengthen screening of close contacts, investigation of infection sources, investigation of clusters of outbreaks, and active detection of people in high-risk areas.

## Data Availability Statement

The original contributions presented in the study are publicly available. This data can be found here: GISAID, the accession numbers can be found in the Supplementary Material.

## Ethics Statement

Written informed consent was not obtained from the individual(s) for the publication of any potentially identifiable images or data included in this article.

## Author Contributions

YS: data curation, investigation, and writing original draft preparation. YZ: data curation, formal analysis, investigation, and resources. ZL: formal analysis, investigation, and methodology. XL and JL: data curation, methodology, and resources. XT, QG, PN, and ZH: data curation and investigation. ZS: methodology, data curation, writing original draft preparation, writing review and editing, and project administration. YT and JW: methodology, data curation, writing review and editing, and project administration. All authors have read and agreed to the published version of the manuscript.

## Funding

This work was supported by the Yantai scientific project (No. 2020YD081), Technological Innovation Project in Shandong Province (No. 2020SFXGFY02-1), National Natural Science Foundation of China (No. 42177418).

## Conflict of Interest

The authors declare that the research was conducted in the absence of any commercial or financial relationships that could be construed as a potential conflict of interest.

## Publisher's Note

All claims expressed in this article are solely those of the authors and do not necessarily represent those of their affiliated organizations, or those of the publisher, the editors and the reviewers. Any product that may be evaluated in this article, or claim that may be made by its manufacturer, is not guaranteed or endorsed by the publisher.

## References

[1] Zhou P Yang XL Wang XG Hu B Zhang L Zhang W. A pneumonia outbreak associated with a new coronavirus of probable bat origin. Nature. 588:E6. 10.1038/s41586-020-2951-z PMC9744119 33199918

[2] Wu F Zhao S Yu B Chen YM Wang W Song ZG. A new coronavirus associated with human respiratory disease in China. Nature. (2020) 579:1–8. 10.1038/s41586-020-2008-3 32015508 PMC7094943

[3] Tan J Liu S Zhuang L Chen L Dong M Zhang J. Transmission and clinical characteristics of asymptomatic patients with SARS-CoV-2 infection. Future Virol. (2020) 15:373–80. 10.2217/fvl-2020-0087

[4] Wu JT Leung K Leung GM. Nowcasting and forecasting the potential domestic and international spread of the 2019-nCoV outbreak originating in Wuhan, China: a modelling study. Lancet. (2020) 395:689–97. 10.1016/S0140-6736(20)30260-9 32014114 PMC7159271

[5] Vacharathit V Aiewsakun P Manopwisedjaroen S Srisaowakarn C Laopanupong T Ludowyke N. SARS-CoV-2 variants of concern exhibit reduced sensitivity to live-virus neutralization in sera from CoronaVac vaccinees and naturally infected COVID-19 patients. medRxiv. [Preprint]. (2021). 10.1101/2021.07.10.21260232

[6] Tian F Tong B Sun L Shi S Zheng B Wang Z. Mutation N501Y in RBD of spike protein strengthens the interaction between COVID-19 and its receptor ACE2. medRxiv. [Preprint]. (2021). 10.1101/2021.02.14.431117

[7] Luan B Wang H Huynh T. Enhanced binding of the N501Y-mutated SARS-CoV-2 spike protein to the human ACE2 receptor: insights from molecular dynamics simulations. FEBS Lett. (2021) 595:1454–61. 10.1002/1873-3468.14076 33728680 PMC8250610

[8] Garcia-Beltran WF Lam EC Denis KS Nitido AD Garcia ZH Hauser BM. Circulating SARS-CoV-2 variants escape neutralization by vaccine-induced humoral immunity. MedRxiv. (2021) 184:2372–83.e9. 10.1101/2021.02.14.21251704 33743213 PMC7953441

[9] Wang WB Liang Y Jin Y Q Zhang J Su JG Li QM. E484K mutation in SARS-CoV-2 RBD enhances binding affinity with hACE2 but reduces interactions with neutralizing antibodies and nanobodies: binding free energy calculation studies. bioRxiv. (2021) 109:108035. 10.1101/2021.02.17.431566 34562851 PMC8447841

[10] Ryzhikov AB Ryzhikov EA Bogryantseva MP Usova SV Danilenko ED Nechaeva EA. A single blind, placebo-controlled randomized study of the safety, reactogenicity and immunogenicity of the “EpiVacCorona” Vaccine for the prevention of COVID-19, in volunteers aged 18-60 years (phase I-II). Russian J Infect Immun. (2021) 11:283–96. 10.15789/2220-7619-ASB-1699

[11] Khateeb J Li Y H Zhang. Emerging SARS-CoV-2 variants of concern and potential intervention approaches. Crit Care. (2021) 25:244. 10.1186/s13054-021-03662-x 34253247 PMC8274962

[12] Scudellari M. How the coronavirus infects cells - and why Delta is so dangerous. Nature. (2021) 595:640–4. 10.1038/d41586-021-02039-y 34321669

[13] McCallum M Czudnochowski N Rosen LE Zepeda SK Bowen JE Walls AC. Structural basis of SARS-CoV-2 Omicron immune evasion and receptor engagement. Science. (2022) 2022:eabn8652. 10.1101/2021.12.28.474380 35076256 PMC9427005

[14] Liu L Iketani S Guo Y Chan JFW Wang M Liu L. Striking antibody evasion manifested by the Omicron variant of SARS-CoV-2[J]. Nature. (2022) 602:676–81. 10.1038/s41586-021-04388-0 35016198

[15] Hui KPY Ho JCW Cheung MC Ng KC Ching RH Lai KL. SARS-CoV-2 Omicron variant replication in human bronchus and lung *ex vivo. Nature*. (2022) 603:715–20. 10.1038/s41586-022-04479-6 35104836

[16] Meng B Ferreira IATM Abdullahi A Goonawardane N Saito A Kimura I. SARS-CoV-2 Omicron spike mediated immune escape and tropism shift. BioRxiv. (2022) 66:15–23. 10.21203/rs.3.rs-1191837/v1 35075452 PMC8786230

[17] Bolger AM Lohse M Usadel B. Trimmomatic: a flexible trimmer for Illumina sequence data. Bioinformatics. (2014) 30:2114–20. 10.1093/bioinformatics/btu170 24695404 PMC4103590

[18] Li H Aligning sequence reads clone sequences and assembly contigs with BWA-MEM. arXiv preprint arXiv: 1303.3997 (2013). 10.48550/arXiv.1303.3997

[19] Tarasov A Vilella AJ Cuppen E Nijman IJ Prins P. Sambamba: fast processing of NGS alignment formats. Bioinformatics. (2015) 31:2032–4. 10.1093/bioinformatics/btv098 25697820 PMC4765878

[20] McKenna A Hanna M Banks E Sivachenko A Cibulskis K Kernytsky A. The genome analysis toolkit: a MapReduce framework for analyzing next-generation DNA sequencing data. Genome Res. (2010) 20:1297–303. 10.1101/gr.107524.110 20644199 PMC2928508

[21] Grubaugh ND Gangavarapu K Quick J Matteson NL De Jesus JG Main BJ. An amplicon-based sequencing framework for accurately measuring intrahost virus diversity using PrimalSeq and iVar. Genome Biol. (2019) 20:1–19. 10.1186/s13059-018-1618-7 30621750 PMC6325816

[22] Zhang M Xiao J Deng A Zhang Y Zhuang Y Hu T. Transmission dynamics of an outbreak of the COVID-19 Delta variant B. 1.617. 2-Guangdong Province, China, May-June 2021. China CDC Wkly. (2021) 3:584. 10.46234/ccdcw2021.148 34594941 PMC8392962

[23] Brief TA. European Centre for Disease Prevention and Control. Emergence of SARS-CoV-2 B.1.617 variants in India and situation in the EU/EEA-11. (2021).

[24] Campbell F Archer B Laurenson-Schafer H Jinnai Y Konings F Batra N. Increased transmissibility and global spread of SARS-CoV-2 variants of concern as at June 2021. Euro Surveill. (2021) 26:2100509. 10.2807/1560-7917.ES.2021.26.24.2100509 34142653 PMC8212592

[25] Lucey M Macori G Mullane N Sutton-Fitzpatrick U Gonzalez G Coughlan S. Whole-genome sequencing to track severe acute respiratory syndrome coronavirus 2 (SARS-CoV-2) transmission in nosocomial outbreaks. Clin Infect Dis. (2021) 72:e727–35. 10.1093/cid/ciaa1433 32954414 PMC7543366

[26] Safdar N Moreno GK Braun KM Friedrich TC O'Connor DH. Using virus sequencing to determine source of SARS-CoV-2 transmission for healthcare worker. Emerg Infect Dis. (2020) 26:2489. 10.3201/eid2610.202322 32758345 PMC7510721

[27] Taylor J Carter RJ Lehnertz N Kazazian L Sullivan M Wang X. Serial testing for SARS-CoV-2 and virus whole genome sequencing inform infection risk at two skilled nursing facilities with COVID-19 outbreaks-Minnesota, April-June 2020[J]. Morbid Mortal Wkly Rep. (2020) 69:1288. 10.15585/mmwr.mm6937a3 32966272 PMC7498172

[28] Paltansing S Sikkema RS de Man SJ Koopmans MPG Munnink BO de Man P. Transmission of SARS-CoV-2 among healthcare workers and patients in a teaching hospital in the Netherlands confirmed by whole-genome sequencing. J Hosp Infect. (2021) 110: 178–83. 10.1016/j.jhin.2021.02.005 PMC7869749 33571558

[29] Chan ER Jones LD Redmond SN Navas ME Kachaluba NM Zabarsky TF. Use of whole-genome sequencing to investigate a cluster of severe acute respiratory syndrome coronavirus 2 (SARS-CoV-2) infections in emergency department personnel. Infect Control Hosp Epidemiol. (2021) 5:1–3. 10.1017/ice.2021.208 33941299 PMC8144813

[30] Pedro N Fernandes V Cavadas B Guimarães JT Barros H Tavares M. Field and molecular epidemiology: how viral sequencing changed transmission inferences in the first Portuguese SARS-CoV-2 infection cluster. Viruses. (2021) 13:1116. 10.3390/v13061116 34200621 PMC8226748

[31] Pachetti M Marini B Benedetti F Giudici F Mauro E Storici P. Emerging SARS-CoV-2 mutation hot spots include a novel RNA-dependent-RNA polymerase variant. J Transl Med. (2020) 18:1–9. 10.1186/s12967-020-02344-6 32321524 PMC7174922

